# Taking the Operant Paradigm into the Field: Associative Learning in Wild Great Tits

**DOI:** 10.1371/journal.pone.0133821

**Published:** 2015-08-19

**Authors:** Julie Morand-Ferron, Steven Hamblin, Ella F. Cole, Lucy M. Aplin, John L. Quinn

**Affiliations:** 1 Department of Biology, University of Ottawa, Ottawa, Canada; 2 Edward Grey Institute, Department of Zoology, University of Oxford, Oxford, United Kingdom; 3 School of Biotechnology and Biomolecular Sciences, University of New South Wales, Sydney, Australia; 4 School of Biological, Earth and Environmental Sciences, University College Cork, Cork, Ireland; University of Guelph, CANADA

## Abstract

Associative learning is essential for resource acquisition, predator avoidance and reproduction in a wide diversity of species, and is therefore a key target for evolutionary and comparative cognition research. Automated operant devices can greatly enhance the study of associative learning and yet their use has been mainly restricted to laboratory conditions. We developed a portable, weatherproof, battery-operated operant device and conducted the first fully automated colour-associative learning experiment using free-ranging individuals in the wild. We used the device to run a colour discrimination task in a monitored population of tits (Paridae). Over two winter months, 80 individuals from four species recorded a total of 5,128 trials. Great tits (*Parus major*) were more likely than other species to visit the devices and engage in trials, but there were no sex or personality biases in the sample of great tits landing at the devices and registering key pecks. Juveniles were more likely than adults to visit the devices and to register trials. Individuals that were successful at solving a novel technical problem in captivity (lever-pulling) learned faster than non-solvers when at the operant devices in the wild, suggesting cross-contextual consistency in learning performance in very different tasks. There was no significant effect of personality or sex on learning rate, but juveniles’ choice accuracy tended to improve at a faster rate than adults. We discuss how customisable automated operant devices, such as the one described here, could prove to be a powerful tool in evolutionary ecology studies of cognitive traits, especially among inquisitive species such as great tits.

## Introduction

Cognition–the processes by which animals collect, store and use information from their environment [1]—has traditionally been studied in captivity, using either lab-raised or wild-caught animals. This allows the control of external factors that could impact on performance during tests, and trials can be administered to targeted subjects on experimenter-defined schedules. However, more recently researchers have begun to develop cognitive assays that can be conducted in the wild, with free-ranging individuals interacting with devices or being tracked in their natural environment [2,3,4,5,6]. This has the advantage of reducing stress linked with capture and housing, and therefore avoiding potential problems associated with interactions between individual stress responses and cognitive performance [7,8]. Moreover, performance is then expressed in a context where the costs and benefits of cognitive processes are consistent with those normally experienced by individuals, with trials occurring under unconstrained group size, habitat complexity, time of day, etc. Combining field experiments and captive assays thus is a valuable approach that could help to achieve both ecological relevance and experimental control, respectively. This integrated approach has been applied for social learning [9,10] and innovative problem solving [11,12,13,14]; here we aim to develop this approach further for associative learning.

Associative learning—a behavioural modification following reinforcement, based on associations between stimuli, or between stimuli and responses–has been found in all bilateral animals tested to date [15,16]. This conserved cognitive process is key to the development of several behaviours, including those involved in foraging [4,17], predator avoidance [18,19], and reproduction [20,21]. Associative learning is therefore an important focus for evolutionary and comparative cognition research [16,22,23].

Several devices and protocols for studying learning and memory have been used in the field, including artificial flowers [5,24,25], differently-coloured feeders [26], and mazes [2,27]. However these studies have often required the experimenter to be present in the field during the presentation of experimental cues and assays. This may influence the behaviour of animals, and is likely to considerably limit the amount of data that can be collected. Automated operant conditioning devices offer a solution to these limitations [28,29]. In operant conditioning experiments, subjects learn to exhibit a behavioural response upon detecting a stimulus (e.g. light or sound) that predicts reinforcement or punishment. However for technical, conceptual and logistic reasons the use of Skinner boxes has traditionally been restricted to laboratory settings (i.e. permanent chambers linked to a central computer).

Here we report on a new portable operant research device that can be used in both field and captive conditions. The device functions as an “inverted”, free operant conditioning chamber, in which subjects that visit voluntarily can proceed to learning trials via an automated process. This approach builds upon earlier work on learning in flocks of free-ranging pigeons [30,31] and socially-housed monkeys [32,33]. In contrast to most operant systems, our device is battery-operated and can be fitted within a 30 x 20 x 30 cm space, allowing its use in the wild away from mains electrical power (see also[34]). It also features a simple technical innovation that removes the need to engage in shaping of the operant response (e.g. [26,35]), i.e. clear response keys containing food that act as an attractant for free-ranging subjects, but can never be taken or depleted.

This presentation of a free-access cognitive testing device in natural settings could however lead to sampling bias, if some individuals are preferentially visiting and using the device [36,37]. Experimental protocols involving novel objects may elicit avoidance in individuals with neophobic personality types [38], or by those that have better access to other resources (e.g. adults compared to juvenile [13]). For instance, individuals that are more active in novel room assays(i.e. “fast explorers” as opposed to “slow explorers” [39]) could be more likely to encounter, contact, and use the devices, due to increased activity rates [37] and a higher propensity to take risks [39,40,41]. It is therefore an important step in the development of field studies on animal cognition to examine the composition of individuals participating in the experiments. Here we take advantage of knowledge derived from the marked population of parids in our long-term study population to assess species, age, sex and personality (exploration) biases in the sample of birds contributing to our wild associative learning experiment.

Empirical evidence for the existence of individual differences in cognitive performance in various cognitive domains has accumulated in the past years [42,43], and have recently been examined in the context of evolutionary ecology studies in natural populations (reviewed in [44, 45]). While there is some evidence for individual differences in associative learning performance within natural populations [26,46,47,48,49,50], little is known about the consistency of these differences across ecological contexts or their impact on life-history traits and fitness in natural populations [44].

Here we examine the link between problem-solving performance at a novel foraging task in captivity [51,52] and learning rate at operant devices in the wild. Though yet to be shown definitively, success at the captive lever-pulling task has been suggested to be mediated by operant learning [52,53]. More accurate colour-associated learning in the wild by those solving problems in captivity would suggest that individual differences in learning performance are robust to drastic variation in the socio-ecological context and the type of learning involved (i.e. operant learning for a motor task vs. colour-based discrimination). Moreover, we examine the effects of sex and age on learning rate, and test the hypothesis that fast explorers are faster at forming simple behavioural associations [47,54,55] and thus at learning an association between colour and food rewards.

Our main goals were therefore to; (1) develop and test an automated operant device and protocol in field conditions; (2) determine any species, sex, age class, or exploration biases in the population of individuals that landed at the devices and recorded trials (i.e. “sampling biases”); (3) compare learning rates of successful and unsuccessful problem-solvers; and (4) to examine individual determinants (sex, age, and exploration score) of learning rate in the wild. We used automated data outputs to examine the sequence of choices made by marked individuals in a simple colour association test. The learning test consisted of a simultaneous discrimination procedure, in which pecking the red key once was rewarded 100% of the time (fixed ratio 1 schedule), and pecking the yellow or green keys was never rewarded. The position of colour cues was varied pseudo-randomly between trials to ensure that birds learnt colour rather than spatial position as a predictor of reward.

## Material and Methods

### Study site and subjects

The study took place in Wytham Woods, Oxfordshire, UK, from 5 January to 3 March 2012. In the context of another study targeting mixed-species flocks of tits in Wytham, great, blue (*Cyanistes caeruleus*), coal (*Periparus ater*), and marsh tits (*Poecile palustris*) were caught in mistnets, each fitted with a PIT (passive integrated transponder) tag [56,57] leg-band in addition to the standard metal leg-ring from the British Trust for Ornithology. All birds were sexed and aged (juvenile/adult) based on plumage [58]. While any bird could potentially visit the operant devices, only individuals equipped with a PIT-tag could be detected when landing on the device; our target sample therefore comprised marked individuals from these four tit species. The proportion of marked great tits in Wytham was estimated at 90% [59].

Species, sex and age composition of the overall population was extracted from a grid of 65 sunflower feeders fitted with RFID antennae that collected spatio-temporal flocking data throughout Wytham woods from December 2011 to March 2012 [60,61]. Given that food was freely available throughout the wood, and that individuals could not dominate feeders, it is assumed that any bias in visitations to feeders was negligible [62]. Secondly, a personality trait, exploration behaviour of a novel environment, was assayed on great tits taken into captivity over 24h between 2007 and 2012. Exploration score captures the behaviour of individuals when placed in a novel room and is calculated using the number and duration of flights, number of hops and the areas of the room utilized during an 8 minute assay [63]. The final score is generated using the first component from a principal component analysis on all of these measures (see [63] for complete methodological details). This trait has been demonstrated to be heritable, is linked to competitive ability, promiscuity and social behaviour in the wild, and is associated with variation in parental care, dispersal, and reproductive success in this population [52,59,63,64,65,66]. Finally, as per another study [52], problem-solving performance was assessed in isolation in captivity the evening and morning preceding the exploration test; birds were exposed to inaccessible waxworms in a clear Perspex tube upon their housing following capture in Wytham woods [52]. To obtain the waxworms, a bird had to pull out the lever, which caused the worms to fall in the receptacle at the bottom of the tube. While nearly all birds collected the free waxworm deposited in the receptacle at the beginning of the trial, only 44% solved the lever-pulling problem (see [51] for details). Problem-solving assays were run in the autumn and winter of 2007 to 2012, and have been shown to predict competitive ability, parental provisioning behaviour and several reproductive life history traits linked with fitness [52,67]. All birds were released at their point of capture in the woods after a short captivity period (24h to 5 days).

### Portable operant devices

**Fig 1:**
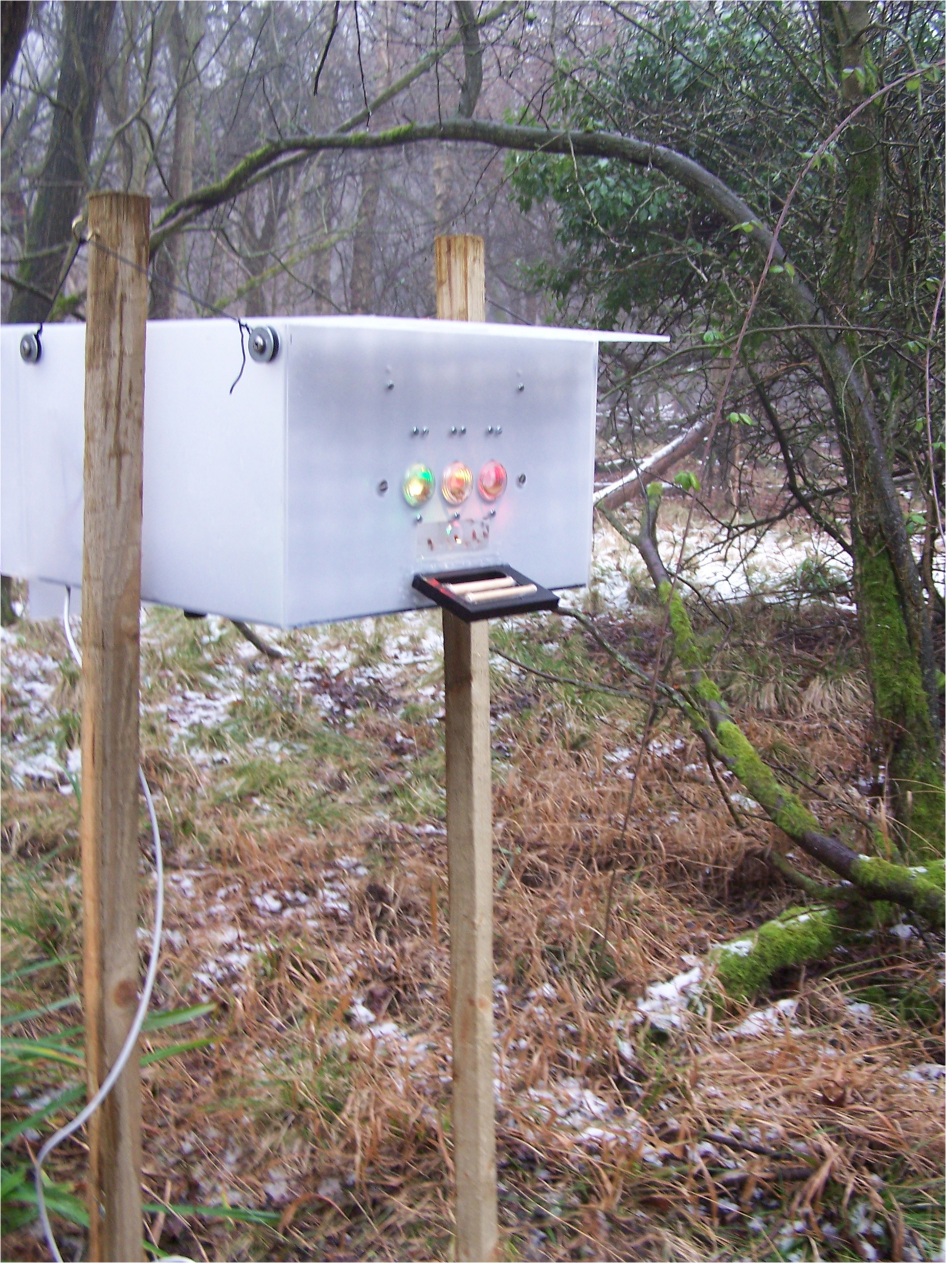
One of the portable operant devices installed in Wytham Woods, Oxford, UK. The weatherproof external casing is made of opaque Perspex, while the three response keys and the section of the front panel comprising the feeding hole are transparent. The perch (bottom of the front panel) is equipped with an antenna that relays information about the unique PIT-tag combination of marked individuals to the printed circuit board located within the device.

We used five identical operant devices for this experiment (Fig 1 and S1 Fig; see also S1 Text and S1–S3 Movies). Each device was composed of (i) weatherproof (Perspex) external casing; (ii) a printed circuit board (PCB); (iii) three response keys; (iv) a motor-activated feeding wheel; and (v) a PIT-tag detection system. The external casing had two hooks on each side so that the device could be installed on wooden stakes planted in the ground (~1.5 m high). The PCB (“Darwin board” designed by Stickman Technologies Inc., UK) receives information from the PIT tag detection system and the response keys, and determines the state of the response keys and of the motor activating the feeding mechanism. The front panel of the operant device was freely available to any wild individual and displayed three response keys located 1 cm from each other, and 1.5 cm above a central feeding hole by which only one reward could be taken. Each key was made up of a circular, transparent plastic container (1.5 cm diameter) held in place in a hole on the front panel by elastics installed within the device, such that even a very light pressure would break a light beam and convey information to the PCB that a choice had been made.

To elicit pecking at the keys without having to shape this response through successive steps (e.g. [35]), we placed four live mealworms (*Tenebrio molitor*) inside each transparent response key. These were visible to a bird landing on the device but could never be taken. The learning program instructed each response key to light up either red, yellow or green with Red-Green-Blue light-emitting diodes, while not using the same colour in the same location more than twice in a row (i.e. a pseudo-random colour sequence to avoid the possibility of spatial learning). To obtain a food reward, individuals had to press the response key that was lit up red (fixed ratio 1 schedule), thereby activating the feeding wheel (<1s) and returning the program to its initial state after 2s. Pressing yellow or green led to no reward and an immediate delay of 15s before a new trial would start. This delay acted as a punishment, preventing birds that made an incorrect choice from immediately beginning another trial. We chose red as the rewarded colour because it is a common aposematic coloration and was expected to be a non-preferred option based on previous experiments in birds [68,69,70]. The observed proportion of initial pecks to the red option did not differ significantly from the random expectation of 33.3% (N = 67, one-sample t-test: t = 0.968, d.f. = 66, p = 0.337). We used two non-rewarding colours instead of one to reduce the probability of exhibiting correct choices randomly. The feeding wheel was made of transparent Perspex and featured 48 wells, each 0.8 cm deep and baited with one half-mealworm. Mealworms are a highly preferred food item for tits, particularly compared with the food available in Wytham’s winter environment, which is mainly seed-based (e.g. beechmast; [71]). Finally, once the 48 rewards were depleted, operant devices turned themselves off.

The identity of PIT-tagged individuals landing at the device was relayed to the PCB by an antenna located in the perch (Dorset Identification, NL). This perch was a horizontal plane of 10cm x 5cm x 1cm and was small enough to hold only one bird at once, but large enough to give access to all three keys on the front panel. The whole system for each device was powered by a 12V sealed lead-acid battery; a 12Ah battery was enough to power a device for at least three days in winter conditions. When the device was powered on (i.e. almost continuously over the experimental period for each location), all keys were illuminated and the timing of visits and PIT combination, as well as the timing and colour choice for each peck, were recorded automatically on an output file stored on a SD card. A visit recorded the time when a PIT-tagged bird landed on the device, whether it pecked at the keys or not (“visit”). A “trial” was defined as a key peck when the keys would be illuminated with colours and a choice had thus been made (see S1 Text).

### Field protocol

Operant devices were deployed at four locations across Wytham Woods. Sites featured an open area with nearby tree cover from which the birds could come and perch on the devices. We first baited each location for several days with a regular multi-access feeder containing sunflower seeds, before installing either two (N = 2 locations) or three operant devices (N = 2 locations) 1m from tree cover, 3 to 8 m from each other, and placing a few mealworms on the roof of each device. We used two to three devices per location to reduce monopolisation of devices by dominant individuals. We then activated the operant devices (see below) and allowed the sunflower feeder to be depleted, refilling it bi-weekly until birds started to peck regularly at the operant devices. Devices were left in place for 26, 18, 21 and 11 days, respectively at each location; devices were removed when depletion rate became high, indicating that no new birds were likely to be given the chance to learn. During this period we changed batteries every 3 days and refilled rewards within devices when they were depleted (no more than twice per day).

### Statistical analysis

We examined species, sex, age class, problem-solving performance and exploratory personality biases among birds that landed at the devices at least once, or registered at least one trial (key peck). The proportion of different species, sexes, age classes, and of solvers (vs. non-solvers) visiting the devices and recording trials were compared with the proportion of birds not visiting the devices or recording trials in the wider Wytham woods population using a Chi-square test with Yates’s continuity correction. Mean exploration score in these groups was compared using two-group t-tests.

We assessed colour-based associative learning by testing for improvement in colour choice accuracy over successive trials by individual birds. Because the number of trials was different for all birds, we scaled trial number by the highest number of trials recorded by any individual (n = 700 trials). We used generalised linear mixed models (GLMM, function “glmer”) with correct (red = 1) vs. incorrect (yellow, green = 0) choice as the response variable, and individual identity as a random intercept. A positive and significant fixed term for trial number would be evidence for learning of the colour-based association. A significant interaction between the random term for ID and trial number (i.e. significant random slope) would be evidence for individual differences in learning rates, and was assessed using the log-likelihood ratio test (LRT) [72,73,74]. Finally, we examined the effect of problem-solving success, age class, sex, and exploration personality score on the rate of learning by testing for significant interactions between each of these terms and trial number. All other individual variables, as well as site and inter-trial interval (log-transformed) were controlled for as fixed factors. Each model included significant random terms (i.e. intercept and slope). Results were qualitatively the same when other individual predictors were excluded (not shown). All analyses were conducted in R version 3.2.0 [75].

### Ethics statement

Work was subject to review by the Department of Zoology ethical committee, University of Oxford. All work adhered to UK standard requirements and was carried out under Natural England licence 20114732. Field work took place in Wytham Woods (lat 51°46’N, long 1°20’W), private land that belongs to the University of Oxford; for permission contact the Conservator, Nigel Fisher. No endangered or protected species were involved in the study.

## Results

### Which species used the free-operant devices?

We recorded 7,480 visits to the operant devices by 144 PIT-tagged birds (Table A in S2 Text). Eighty of these birds (55.6%) registered at least one trial by pecking at one of the three keys (range 1–700 trials per individual), with 5,128 trials recorded across all birds. Great tits recorded the highest number of trials (N = 5,086), followed by blue tits (N = 32), marsh tits (N = 9) and coal tits (N = 1; Table A in S2 Text). The proportion of great tits having recorded at least one visit and at least one trial was higher than the proportion among the four Parid species wintering in Wytham (Table 1; visits: Χ^2^ = 31.3, d.f. = 1, p < 0.001; trials: Χ^2^ = 68.2, d.f. = 1, p < 0.001). On average, great tits collectively recorded 66.9 trials per day per site, with this value reaching an average of 698 trials per site on the last complete day of data collection (mean ± s.e. number of trials per individual great tit = 75.9 ± 19.4; See also Table A in S2 Text).

**Table 1:** The numbers (and percentages of species totals) in a marked population of Paridae spp. wintering in Wytham woods in 2011–2012 (Wytham). Statistics are shown for individuals that visited devices at least once (Visited), and for those that had pecked at least one key (Pecked).

*Species*	*Wytham*	*Visited*	*Pecked*
Blue tit	1534 (54.2%)	43 (29.9%)	8 (10.0%)
Coal tit	103 (3.6%)	3 (2.1%)	1 (1.3%)
Great tit	1061 (37.5%)	88 (61.1%)	67 (83.8%)
Marsh tit	134 (4.7%)	10 (6.9%)	4 (5.0%)
Totals	2832 (100%)	144 (100%)	80 (100%)

### Sex, age class and personality biases in use of the devices by great tits

The sex ratio of great tits having recorded at least one visit and at least one trial was not significantly different from the sex ratio in the wintering population (Table 2; visits: Χ^2^ < 0.01, d.f. = 1, p = 0.976; trials: Χ^2^ = 0.003, d.f. = 1, p = 0.956). However, the proportion of juveniles having recorded at least one visit and at least one trial was greater than the corresponding proportion in adults (Table 3; visits: Χ^2^ = 8.92, d.f. = 1, p = 0.003; trials: Χ^2^ = 8.61, d.f. = 1, p = 0.003). The proportion of problem-solvers who registered visits or trials at the devices did not differ from that of non-solvers (visits: Χ^2^ = 0.57, d.f. = 1, p = 0.451; trials: Χ^2^ = 0.006, d.f. = 1, p = 0.941). Mean exploration score did not differ between great tits that had (and had not) recorded at least one visit at the device (-0.326 ± 0.048 vs. -0.319 ± 0.067), or amongst those that had (and had not) pecked at least one key (-0.033 ± 0.032 vs. -0.276 ± 0.073; visits: t = -1.0, d.f. = 51.7, p = 0.924; trials: t = -0.71, d.f. = 39.5, p = 0.479).

**Table 2:** The number (and percentage of species totals) of great tits of each sex in a marked population wintering in Wytham woods in 2011–2012. Statistics are shown for individuals that visited devices at least once (Visited), and for those that had pecked at least one key (Pecked). Individuals that had been marked as chicks and not sexed *a posteriori* (N = 3), and those not recorded on the regular grid of feeders (N = 1; see Methods for details) were not included in the analyses.

*Sex*	*Wytham*	*Visited*	*Pecked*
Female	478 (50.4%)	43 (51.2%)	34 (51.5%)
Male	471 (49.6%)	41 (48.8%)	32 (48.5%)
Totals	949 (100%)	84 (100%)	66 (100%)

**Table 3:** The number (and percentage of species totals) of each of the two age classes of great tits in a marked population wintering in Wytham woods in 2011–2012. Statistics are shown for individuals that visited devices at least once (Visited), and for those that had pecked at least one key (Pecked). Individuals not recorded on the regular grid of feeders (N = 1; see Methods for details) were not included in the analyses.

*Age class*	*Wytham*	*Visited*	*Pecked*
Juvenile	501 (49.4%)	58 (66.7%)	46 (68.5%)
Adult	514 (50.6%)	29 (33.3%)	21 (31.3%)
Totals	1014 (100%)	87 (100%)	67 (100%)

### Colour-based associative learning in the wild

**Fig 2:**
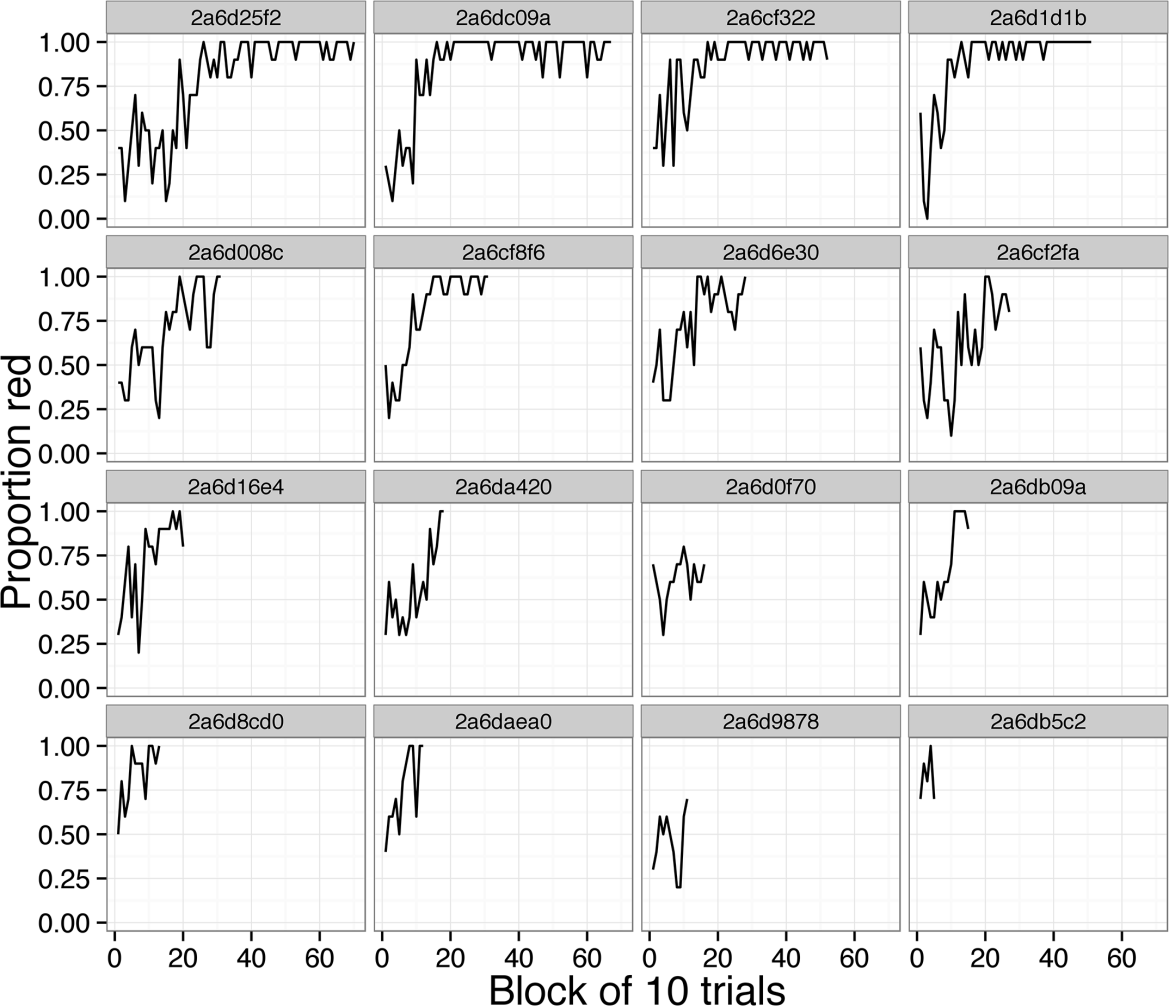
Proportion of pecks to the red key calculated over blocks of 10 trials. One panel per individual (N = 16 birds; only individuals having recorded 50 trials or more are shown).

**Fig 3:**
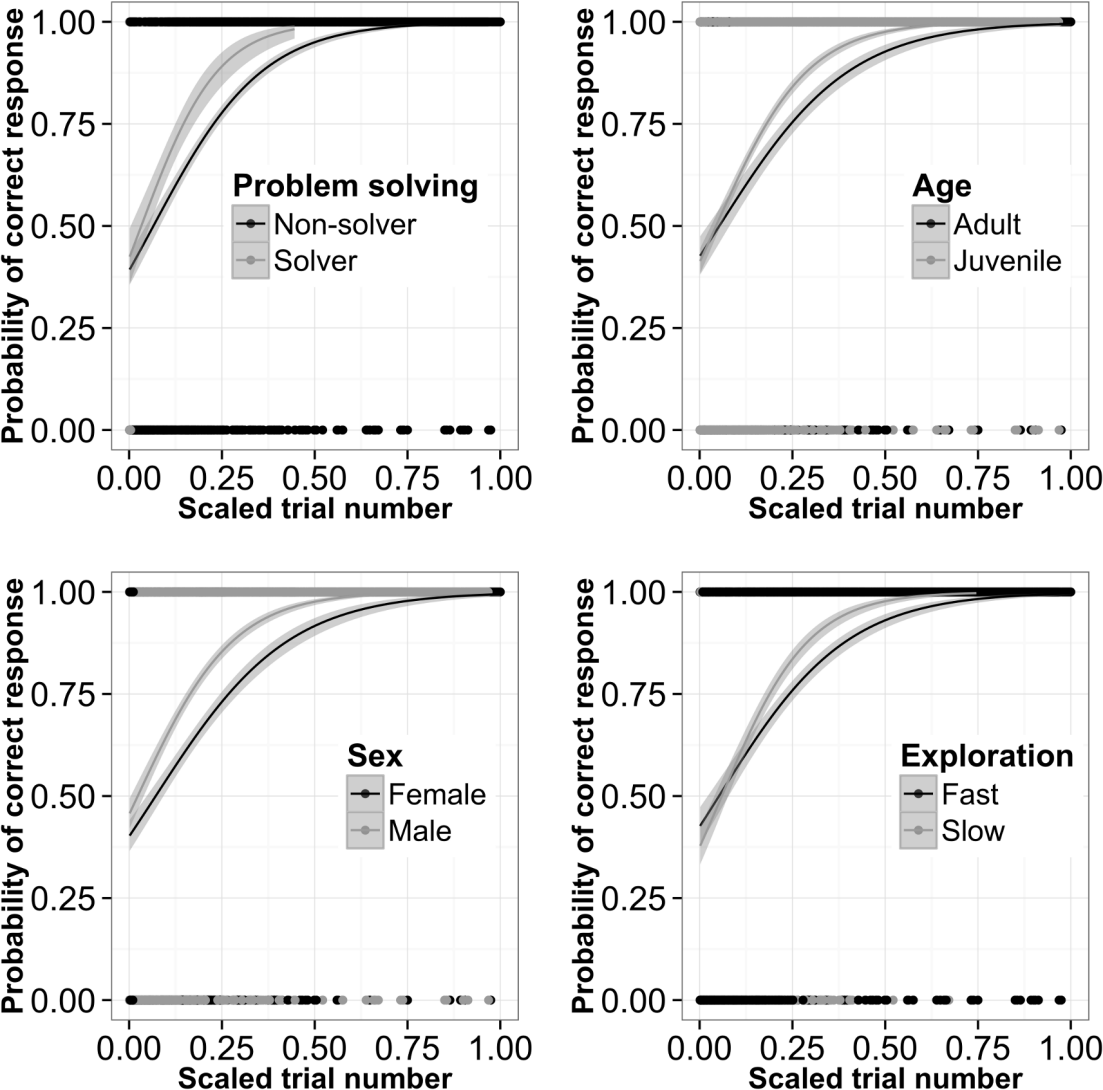
Probability of correct responses (red choices) over successive trials (scaled trial number; see text for details) in free-ranging great tits, with 95% confidence intervals around the GLM logistic curve. (a) non-solvers vs. solvers; (b) adults vs. juveniles; (c) females vs. males; and (d) fast explorers vs. slow explorers. Note that exploration score was analysed as a continuous variable but was split into 50% slower birds vs. 50% fastest explorers for illustration purposes.

Individual great tits (N = 67) improved their colour choice accuracy over successive trials in the field (trial number: Χ^2^ = 563.2, d.f. = 1, p < 0.001; see Fig 2 for examples). Furthermore, there were significant individual differences in learning rate over trials (Χ^2^ = 29.1, d.f. = 1, p < 0.001). We explored determinants of this individual variation in learning performance for great tits of known sex, age class, personality and problem-solving success (N = 21). We found that: (i) solvers learned faster than non-solvers (Fig 3A; Χ^2^ = 5.3, d.f. = 1, p = 0.021); (ii) juveniles showed a marginally non-significant tendency to learn faster than adults (Fig 3B; Χ^2^ = 3.5, d.f. = 1, p = 0.061); but there was no effect of (iii) sex (Fig 3C; Χ^2^ = 0.4, d.f. = 1, p = 0.547), or of (iv) exploration score on learning rate in the field (Fig 3D; Χ^2^ = 0.97, d.f. = 1, p = 0.326) (see also Tables B-E in S2 Text).

## Discussion

We developed a portable, automated operant device and tested a research protocol for the measurement of associative learning in free-ranging songbirds. Wild parids landed on the free-operant device, and great tits could learn to obtain food rewards by pecking at the red-lit key, but not at the green or yellow-lit keys. Our devices were therefore successful in administering learning trials without the need to shape or train individuals before test trials, or requiring an experimenter to be present during the trials. This is, to our knowledge, the first time a fully-automated colour-associative learning test recording individual behaviour has been administered to free-ranging animals.

The experimental protocol was successful in attracting a large number of free-ranging individuals to the devices: 144 PIT-tagged birds visited the devices within two months of automated field observations. All species were able to register trials at the devices but only great tits proved to be a useful model species for this type of study at Wytham, with 76% of individuals visiting the devices registering at least one trial. It is possible that species differences arose from differences in body size, and that the ca. 100% larger great tit [61,76] found it easier to peck the keys, and faced relatively lower energy costs when doing so. Alternatively they may have been more likely to exhibit a pecking motor action than other species. We suggest that species differences in visit and trial registration at the devices was not due to aggressive monopolisation of the device by great tits because we installed two to three replicates of the devices at each site, and there were long periods when no bird was present at one or several devices.

The sex ratio and mean exploration score of individual great tits using the devices did not differ significantly from the rest of the tagged population, suggesting that the experimental sample was not biased in these respects. It might have been expected that fast explorers should be more likely to encounter the devices due to increased activity rate [37], and more likely to use the device due to lower levels of neophobia and a higher propensity to take risks [39,40,41]. However the long duration of the experiment might have allowed novelty responses to reduce with time, eliminating personality biases in the sampled population, analogously to the process of familiarisation used in captive studies of cognition (e.g. [49]). Juveniles were more prevalent in the experimental sample than in the wintering population in terms of both those that visited the devices and those that engaged in trials. This age bias is consistent with other behavioural studies showing increased participation or persistence by young individuals [13,33,77]. Whether this results from reduced access to other resources due to lower competitive rank, or reduced knowledge about alternative food sources, remains to be determined.

Individual great tits improved gradually over trials in their choice accuracy, providing evidence for colour-based discrimination occurring in the wild. Individuals differed significantly in the rate at which they learned this colour-based association in the field, even when controlling for site and inter-trial interval duration. Birds that were successful at a novel foraging problem (tested in isolation, in captivity) learned the colour association faster than non-solvers once released in the field, which could suggest that individual differences in general associative learning abilities expressed in the novel problem-solving situation and in this colour-based learning test can be stable over time and consistent across contexts. Positive correlations were also observed between innovative problem solving performance and efficiency in motor learning [78,79], as well as associative learning tasks [80] in captive tests on different avian species. Innovative performance was not significantly associated with colour discrimination performance in free-ranging bowerbirds (*Ptilonorhynchus maculates*) but it loaded positively on the first principal component of an analysis including five other cognitive tasks, suggesting that fast problem-solvers also performed well on other cognitive tasks [6]. Between-task consistency in problem-solving performance had already been demonstrated in great tits, with successful lever-pullers being more likely to succeed at a string-pulling task in captivity [51]. Yet few studies have examined the consistency in cognitive performance of the same individuals tested in the wild versus in captivity, and to our knowledge, all have involved similar innovative problem-solving tasks in both contexts [12,13]. The current demonstration of individual consistency when using a reward mechanism that provided only one food item at a time contrasts with partial evidence for consistency in a previous study [13], suggesting that scrounging opportunities or costs may act to reduce cross-contextual consistency in cognitive performance (see also [32]).

Juveniles were more likely to use the devices and showed a marginally non-significant tendency to learn faster than adults. Faster improvement over repeated trials in juveniles cannot be explained by potential differences in temporal patterns of device use because all analyses controlled for inter-trial interval duration. Interestingly, this effect is similar to more rapid learning by young versus older baboons (*Papio papio*) engaged in an automated self-administered associative learning task where individuals took trials at their own pace [81]. The interplay between the effect of voluntary participation (versus forced trials) and individual characteristics of subjects on cognitive performance clearly deserves further investigation. Sex and personality scores did not significantly impact learning rates in this experiment, but a larger sample size would give greater power to detect any such effects in future tests. Finally, the impact of social and ecological factors on cognitive performance (e.g. predation risk, group size [82]) has yet to be examined in this context; automated detection of individuals allows recording and using information on these variables in the natural context [44].

Because trials were not run on individuals in isolation, birds could potentially have learned to peck at the red key by observing successful conspecifics interacting with the device [83,84]. We failed to detect evidence of observational learning in a previous field experiment in the same population using a simpler novel foraging problem and larger sample sizes [13]. However great tits and blue tits are known to rely on social information to find patchy food sources in the wild [85,86], to be able to learn socially from conspecifics in aviary experiments [87,88], and to learn positional cues socially in the wild [89]. Whether birds used social information in the form of cues derived from the foraging activity of others at the devices to increase their probability or frequency of visit (local enhancement, which would not affect choice accuracy) or information about the correct option (observational learning, which could impact learning rates) is currently under investigation. To rule out social information use, a possible modification would be to install an opaque box around the perch so as to limit the availability of visual cues in the field [90].

In conclusion, our operant device has the advantages of being portable, affordable (overall unit price around £700), and capable of automatically running and recording large numbers of trials (up to 698 trials daily per location in this experiment) in individual-based learning assays, with relatively little human intervention in the field. However not all species seem to be responsive to this experimental set-up, with great tits being the only species among the four tit species in Wytham to show participation rates >50%. Because the response keys can be replaced by any other mechanical object that can break the optical beam located behind the front panel, experimenters could potentially use the device with a range of avian or non-avian species, making it a useful tool in comparative cognition research between or within species. The device can be customised by users to administer several types of rewards and tests; for instance multiple schedules of reinforcement can be programmed for the different keys, and reversing the contingencies attributed to each colour would assess reversal learning speed (e.g. [91]). Other aspects of cognition than discrimination could also be examined, including extinction, memory span and accuracy. We hope this work will contribute to the development of integrated evolutionary and comparative research on animal learning by broadening the contexts in which this traditional laboratory equipment can be used.

## Supporting Information

S1 Text Supplementary methods. (PDF)

S2 Text Supplementary results. Total number of visits to the devices, proportion of these visits leading to at least one trial (i.e. key-peck), number of individuals with at least one trial recorded in the field, along with range (minimum-maximum), median and mean ± standard error (s.e.) for total number of trials per individual, for each of the four species (Table A in S2 Text). Results of a binomial GLMM for correct (red) vs. incorrect (green, yellow) choices over successive trials, including a random intercept for individual identity and a random slope for identity over scaled trial number (N = 3470 trials by 21 individuals). This model examines differences in learning slopes over trials in successful problem-solvers vs. non-solvers (Table B in S2 Text). Results of a binomial GLMM for correct (red) vs. incorrect (green, yellow) choices over successive trials, including a random intercept for individual identity and a random slope for identity over scaled trial number (N = 3470 trials by 21 individuals). This model examines differences in learning slopes over trials in adults versus juveniles (Table C in S2 Text). Results of a binomial GLMM for correct (red) vs. incorrect (green, yellow) choices over successive trials, including a random intercept for individual identity and a random slope for identity over scaled trial number (n = 3470 trials by 21 individuals). This model examines differences in learning slopes over trials in females vs. males (Table D in S2 Text). Results of a binomial GLMM for correct (red) vs. incorrect (green, yellow) choices over successive trials, including a random intercept for individual identity and a random slope for identity over scaled trial number (n = 3470 trials by 21 individuals). This model examines differences in learning slopes over trials in relation to exploration score Table E in S2 Text).
(PDF)

S1 Fig Steps to building a portable operant device equipped with PIT recognition system and printed circuit board. (PDF)

S1 Movie Free-ranging great tit registering three successful trials. (WMV)

S2 Movie Free-ranging great tit registering two successful trials. (WMV)

S3 Movie Free-ranging great tit registering two unsuccessful trials. (WMV)

S1 Dataset Wytham great tits. Great tits present in the population in the winter 2012.
(XLSX)

S2 Dataset Trials by great tits. Trials recorded by great tits on the operant boxes.
(XLSX)
