# Supplementary material for: Taking the Operant Paradigm into the Field: Associative Learning in Wild Great Tits
Source: PLoS One. 2015 Aug 19;10(8):e0133821. doi: 10.1371/journal.pone.0133821 (PMC4546055; doi:10.1371/journal.pone.0133821)
Supplement: S1 Text — (PDF) [file pone.0133821.s001.pdf]

Morand-Ferron et al.: Taking the operant paradigm into the field: associative learning in wild great tits

Supplementary Methods

On randomly-chosen days, we installed a video camera 2m in front of a device in order to compare the data that could be extracted from videos with automated data output from the device (we tried to opportunistically cover all devices). We examined (i) record accuracy of pecks on the automated data output, and (ii) detection accuracy of PIT tags by the PIT reader located in the horizontal perch.

We observed 147 pecks (trials) from 10h of video files at the four locations. All 147 pecks (100%) were recorded accurately (correct colour and position) on the output file produced automatically by the device. Most of the time only one contact with the pecking key was enough to turn the key off, thereby indicating that this trial had been recorded, with only nine cases (6.1%) of multiple pecks required to activate the key. The detection of PIT-tagged birds by the antenna located in the horizontal perch and the decoder soldiered onto the PCB (Dorset ID, NL) was not optimal because of the need to provide a horizontal surface for birds to access all three keys. In contrast to this set-up where birds perch on top of the antenna, PIT tags are best detected within the antenna loop. Detection could however be improved by drilling a hole in the middle section of the antenna and placing wooden perches on areas of the antenna were detection accuracy was highest, i.e. avoiding lateral portions of the antenna (see Fig 1 in main text). This allowed recording 118 out of the 147 (80.3%) pecks by PIT-tagged individuals. This detection accuracy could be brought up to 91.2% (134/147) by attributing blank pecks to a PIT-tagged bird if this bird was detected 1s before or 1 s after the peck (i.e. using a ±1 s buffer for detection). Over the course of these observations, we did not detect stealing of food rewards by con- or heterospecifics.

Before the beginning of the experimental period we refined the research protocol by installing devices in different locations than the ones used for actual experiments. Out of the twenty-six individuals who registered at least one peck during pre-trials, only two (7.7%) were also recorded during experimental trials; these two individuals were removed from the final sample.
