# Supplementary material for: Taking the Operant Paradigm into the Field: Associative Learning in Wild Great Tits
Source: PLoS One. 2015 Aug 19;10(8):e0133821. doi: 10.1371/journal.pone.0133821 (PMC4546055; doi:10.1371/journal.pone.0133821)
Supplement: S2 Text — Total number of visits to the devices, proportion of these visits leading to at least one trial (i.e. key-peck), number of individuals with at least one trial recorded in the field, along with range (minimum-maximum), median and mean ± standard error (s.e.) for total number of trials per individual, for each of the four species (Table A in S2 Text). Results of a binomial GLMM for correct (red) vs. incorrect (green, yellow) choices over successive trials, including a random intercept for individual identity and a random slope for identity over scaled trial number (N = 3470 trials by 21 individuals). This model examines differences in learning slopes over trials in successful problem-solvers vs. non-solvers (Table B in S2 Text). Results of a binomial GLMM for correct (red) vs. incorrect (green, yellow) choices over successive trials, including a random intercept for individual identity and a random slope for identity over scaled trial number (N = 3470 trials by 21 individuals). This model examines differences in learning slopes over trials in adults versus juveniles (Table C in S2 Text). Results of a binomial GLMM for correct (red) vs. incorrect (green, yellow) choices over successive trials, including a random intercept for individual identity and a random slope for identity over scaled trial number (n = 3470 trials by 21 individuals). This model examines differences in learning slopes over trials in females vs. males (Table D in S2 Text). Results of a binomial GLMM for correct (red) vs. incorrect (green, yellow) choices over successive trials, including a random intercept for individual identity and a random slope for identity over scaled trial number (n = 3470 trials by 21 individuals). This model examines differences in learning slopes over trials in relation to exploration score Table E in S2 Text). (PDF) [file pone.0133821.s002.pdf]

Morand-Ferron et al.: Taking the operant paradigm into the field: associative learning in wild great tits

Supplementary Tables

Table A. Total number of visits to the devices (“N”), total number of trials (“N”), along with range (minimum-maximum), median and mean ± standard error (s.e.) for total number of trials per individual, for each of the four species.

Species	Visits *N*	Trials *N*	Number of trials per individual *Range*	*Median*	*Mean ± s.e.*
Great tits	7159	5086	1-700	6	75.9 ± 19.4
Blue tits	237	32	1-13	1	4.0 ± 1.7
Marsh tits	78	9	1-6	1	2.3 ± 1.4
Coal tits	6	1	1-1	1	1.0 ± 0.0

Table B. Results of a binomial GLMM for correct (red) vs. incorrect (green, yellow) choices over successive trials, including a random intercept for individual identity and a random slope for identity over scaled trial number (n = 3465 trials by 21 individuals). This model examines differences in learning slopes over trials in successful problem-solvers vs. non-solvers.

Fixed term	X^2	*d.f.*	*p*
Site in Wytham	1.110	3	0.777
Inter-trial interval (log)	2.592	1	0.107
Sex	2.245	1	0.134
Age	0.587	1	0.444
Exploration	7.632	1	0.006
Problem-solving * Trial	5.329	1	0.021

Table C. Results of a binomial GLMM for correct (red) vs. incorrect (green, yellow) choices over successive trials, including a random intercept for individual identity and a random slope for identity over scaled trial number (n = 3465 trials by 21 individuals). This model examines differences in learning slopes over trials in adults versus juveniles.

Fixed term	X^2	*d.f.*	*p*
Site in Wytham	1.418	3	0.701
Inter-trial interval (log)	3.514	1	0.061
Sex	3.261	1	0.071
Exploration	7.623	1	0.006
Problem-solving	0.266	1	0.606
Age * Trial	0.036	1	0.006

Table D. Results of a binomial GLMM for correct (red) vs. incorrect (green, yellow) choices over successive trials, including a random intercept for individual identity and a random slope for identity over scaled trial number (n = 3465 trials by 21 individuals). This model examines differences in learning slopes over trials in females vs. males.

Fixed term	X^2	*d.f.*	*p*
Site in Wytham	1.172	3	0.760
Inter-trial interval (log)	3.293	1	0.070
Age	0.590	1	0.442
Exploration	6.308	1	0.012
Problem-solving	0.010	1	0.919
Sex * Trial	0.363	1	0.547

Table E. Results of a binomial GLMM for correct (red) vs. incorrect (green, yellow) choices over successive trials, including a random intercept for individual identity and a random slope for identity over scaled trial number (n = 3465 trials by 21 individuals). This model examines differences in learning slopes over trials in relation to exploration score.

Fixed term	X^2	*d.f.*	*p*
Site in Wytham	0.802	3	0.849
Inter-trial interval (log)	3.465	1	0.063
Sex	2.264	1	0.132
Age	1.757	1	0.185
Problem-solving	0.321	1	0.571
Exploration * Trial	0.965	1	0.326
