# Supplementary material for: Taking the Operant Paradigm into the Field: Associative Learning in Wild Great Tits
Source: PLoS One. 2015 Aug 19;10(8):e0133821. doi: 10.1371/journal.pone.0133821 (PMC4546055; doi:10.1371/journal.pone.0133821)
Supplement: S1 Fig — (PDF) [file pone.0133821.s003.pdf]

Morand-Ferron et al.: Taking the operant paradigm into the field: associative learning in wild great tits

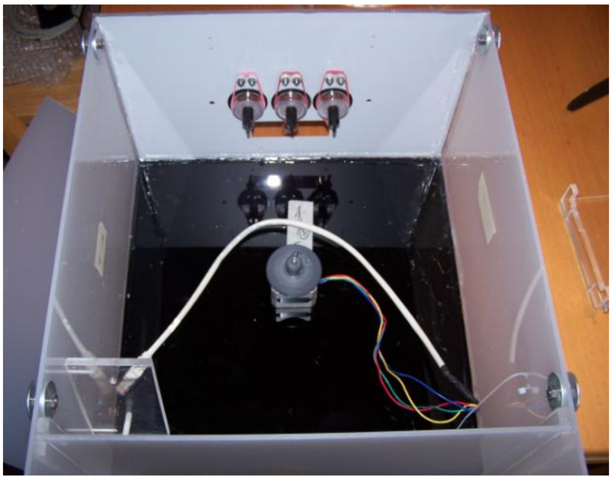

1. In a weatherproof box, install a stepper motor for food delivery. The distance to the front panel must match the diameter of the feeding wheel. The front panel has a central hole for food delivery right below the pecking keys. Each key is held by an elastic and screws afixed above and below the keys (keys: *bead display storage box*, 30x30x17mm).

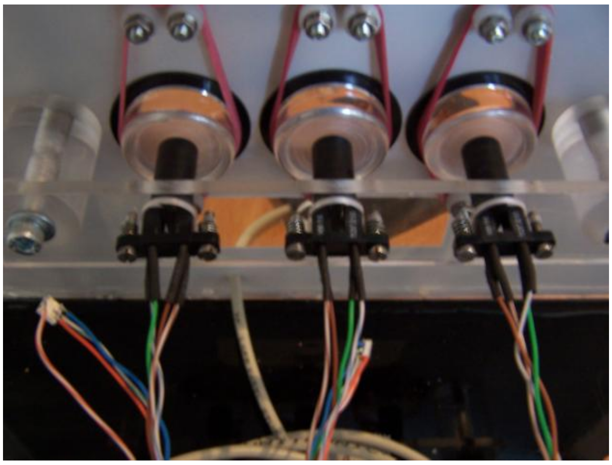

2. Assemble LED-equipped, beam-activated sensors and mount them at an equal distance from the pecking keys. Glue or screw a plastic stick at the back of each key so that this stick is pushed within the range of the sensor when the key is pecked. Glue the LED on the mounting rack, behind each key.

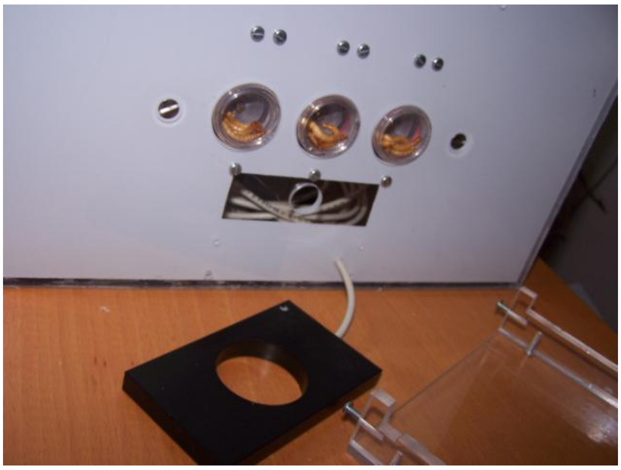

3. Install a passive integrated transponder antenna below the feeding hole on the front panel , connected to the reader located inside the box (here: *ANTC40 and LID665* from Dorset ID, www.dorset.nu). The height of the perch must match the height of the target species. Bait each pecking key with rewards (here: mealworms).

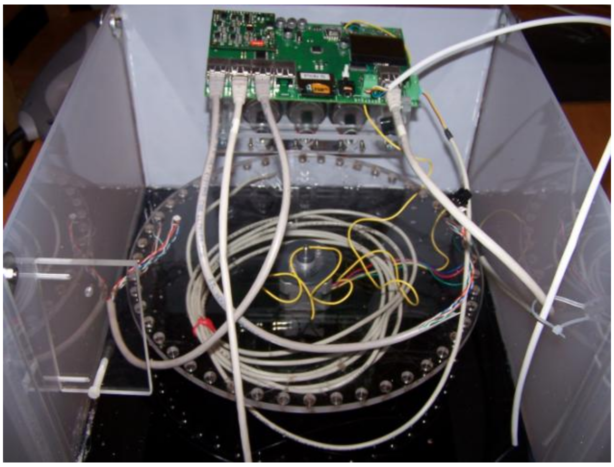

4. Place a feeding wheel on the motor, and the printed circuit board inside the box, at a location that will be accessible once you close the box (here : *Darwin board* from Stickman Technologies, www.sticktech.co.uk). Connect all cables to the board, insert a SD card, and plug the board to a 12V sealed lead-acid battery.

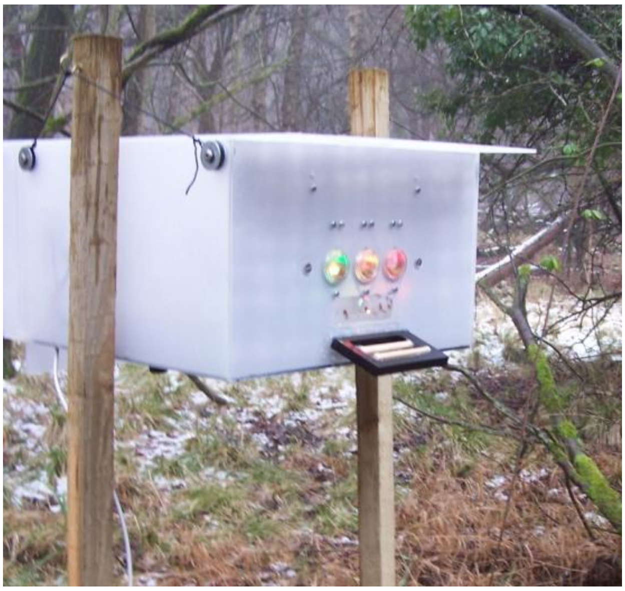

5. Install the operant box onto the ground or onto wooden poles at a height that is appropriate for the target species. Here we used an elongated roof to protect the device from the rain or snow.

Figure A. Steps to building a portable operant device equipped with PIT recognition system and printed circuit board.
